# Evaluation of a Blood Reserve Protocol for Hip Fracture Surgery in the Elderly

**DOI:** 10.1055/s-0044-1785520

**Published:** 2024-09-04

**Authors:** Maurício Rodrigues Miyasaki, Lucas de Quadros Marques, Thiago dos Santos Miranda, André Ruan Ruiz, Karen Barros Parron Fernandez, Bruna Biglia

**Affiliations:** 1 Irmandade da Santa Casa de Londrina, Londrina, PR, Brasil; 2 Instituto de Ensino, Pesquisa e Inovação (IEPI), Irmandade da Santa Casa de Londrina, Londrina, PR, Brasil; 3 Instituto de Hematologia de Londrina, Londrina, PR, Brasil

**Keywords:** blood transfusion, blood typing, hip fractures

## Abstract

**Objective** To identify the predictive factors for the need for transfusion during and after surgery to treat hip fractures in the elderly and to evaluate a protocol to guide the request for blood reserves for surgery.

**Methods** The medical records of 172 elderly patients undergoing surgical treatment for proximal femoral fractures were collected. Data on sex, age, preoperative hemoglobin level, diagnosis, and type of surgery were tested for correlation with blood transfusion. In our sample, we determined the number of units of packed red blood cells reserved, the transfusion rate, and the cross-test:transfusion ratio. We made the same calculations in a hypothetical situation in which the request for blood reserves for our sample followed the criteria of a defined protocol.

**Results** We found a correlation between the American Society of Anesthesiologists (ASA) classification and the occurrence of transfusions, and an inverse correlation between the hemoglobin level on admission and the number of bags transfused. A reserve of 328 units of packed red blood cells was requested for 167 surgeries. Had the proposed protocol been applied, 72 units would have been requested for 61 surgeries.

**Conclusion** We found a correlation regarding the variables ASA classification and preoperative hemoglobin level and the occurrence of transfusion. Applying a proposed protocol to decide between requesting a reserve and only typing for elderly individuals undergoing surgical treatment for hip fracture proved suitable to reduce the number of packed red blood cell reserves.

## Introduction

Hip fractures in the elderly are a public health problem worldwide. As well as being associated with high morbidity and mortality rates, they have a significant financial impact on health systems. 1

Various factors that influence the evolution of these patients are studied, including the importance of multidisciplinary care, 2 the time elapsed between hospitalization and surgery, 3 the problem of previous use of anticoagulants, 4 the types of implants used 5 etc. Among these issues, the management of the use of blood products to treat pre- and postoperative anemia is relevant. Blood transfusion is part of the therapeutic arsenal in the clinical care of elderly individuals with hip fractures. Still, it is a procedure associated with risks such as increased rates of postoperative infection 6 and increases in the cost of treatment.

The criteria to indicate transfusion of packed red blood cells in elderly patients undergoing surgical treatment for hip fractures are still poorly defined. There are liberal protocols that indicate transfusion when hemoglobin levels fall below 10 g/dL, and restrictive protocols that do not indicate transfusion unless the hemoglobin level falls below 8 g/dL or the patient has symptoms related to anemia. There is evidence that liberal protocols do not present advantages in terms of mortality, functional recovery, and complications compared to restrictive protocols, indicating that the restrictive protocol may be considered the better option. 7

Two other critical questions are in which situations and how many units of packed red blood cells should be requested for patients undergoing surgery. Requesting a reserve of packed red blood cells involves the performance of three procedures by the blood bank: ABO and Rh typing, irregular antibody screening (IAS), and crossmatch tests between donor and recipient samples. 8 9 Therefore, each blood bag reserve requested represents a financial impact and generates a workload for the blood bank.

Not all patients undergoing surgical treatment for hip fractures need to receive blood component transfusions; some studies mention an incidence of around 30% to 40%. 7 10 11 12 Therefore, knowing the predictive factors regarding the need for transfusion of packed red blood cells can guide the request for blood before surgery. For patients at low risk of transfusion during and after surgery, requesting only the typing without requesting a reserve would represent a significant advance in managing scarce resources such as blood products.

The present study aimed to identify predictors of the need for transfusion during and after surgery to treat hip fractures in the elderly and to evaluate a protocol to guide the request for blood reserves for surgery.

## Materials and and Methods

### Ethical procedures

The institutional Ethics in Research Committee approved this project under CAAE number 70915423.1.0000.0099.

### Design and Study Population

For data collection, this retrospective cohort was developed using the electronic medical records of patients older than 60 years of age who underwent surgical treatment for proximal femoral fractures at our institution between January 1st and December 31st, 2021. Our institution's Hematology Institute provided the information regarding request and use. Patients with high-energy trauma fractures and those undergoing percutaneous fixation with cannulated screws were excluded.

### Data collection

Data on sex, age, preoperative hemoglobin level, diagnosis, and type of surgery were tested for correlation with blood transfusion.

The transfusion index (TI) and the crossmatch test:transfusion ratio (C:T) were calculated. 13 14 The TI measures the average number of blood units used per procedure, and values lower than 0.5 indicate that the routine request for blood reserves is not necessary for the procedure. The C:T is the ratio between the number of blood units requested and the number of units transfused, and it measures the efficiency of blood requests. Values above 2.5 indicate excessive requests. 13

Based on these criteria, Khan et al. 14 proposed a protocol to request blood for the surgical treatment of hip fractures ( Table 1 ). We included the data obtained in our sample in this protocol to verify its applicability in our casuistry by evaluating the results obtained for the C:T, the number of transfusions carried out without requesting a reserve, and the IT for each hemoglobin level interval in the protocol.

**Table 1:** Blood request protocol for hip fracture surgeries proposed by Khan et al. 14

Hemoglobin level (g/dL)	Blood requests
≥ 11	Typing
9.0–10.9	Reservation of 1 unit
8.0–8.9	Reservation of 3 units
7.0–7.9	Reservation of 4 units
≤ 6.9	Clinical assessment

### Statistical Analysis

For the statistical analysis, we used the PASW Statistics for Windows (SPSS Inc., Chicago, IL, United States), version 18.0, with a 95% confidence interval and 5% significance level ( *p* < 0.05) for the tests applied. The Shapiro-Wilk test was used to verify the normality of the data, and the paired Student *t* -test was used to compare the pre- and postoperative hemoglobin values in the two fracture groups (pertrochanteric and femoral neck).

## Results

Data was collected from 172 medical records of patients who met the study requirements ( Table 1 ). Our sample consisted of 118 female and 54 male patients, with a mean age of 80.2 years. The diagnosis was of pertrochanteric fracture in 100 cases and of femoral neck fracture in 72.

**Fig. 1:**
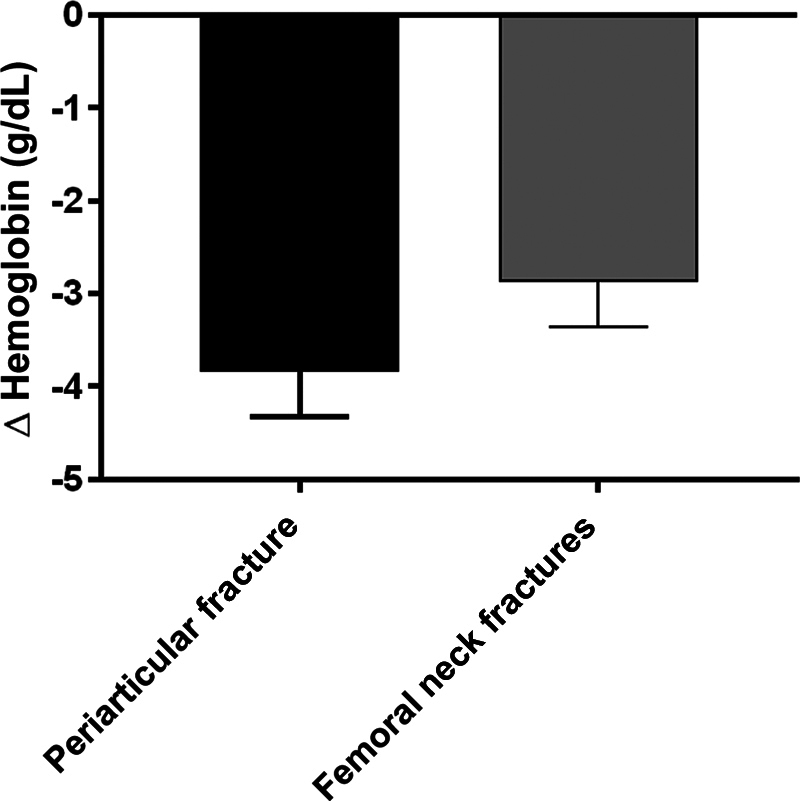
Hemoglobin variation (g/dL) in the different surgical groups.

The mean preoperative hemoglobin level was of 11.44 g/dL; postoperatively. it was of 9.9 g/dL. The preoperative hemoglobin levels were of 11.0 g/dL and 11.7 g/dL respectively for pertrochanteric and femoral neck fractures. In the classification of the American Society of Anesthesiologists (ASA), 3 patients were classified as ASA 1, 128, as ASA 2, 38, as ASA 3, and 3, as ASA 4. After surgery, the average was of 8.9 for pertrochanteric and 9.2 for femoral neck fractures. Although a reduction in the patients' hemoglobin was observed postoperatively (paired *t* -test; *p* = 0.0001), there were no statistical differences between the fracture groups (unpaired *t* -test; *p* = 0.17, Fig. 1 ).

Of those diagnosed with a pertrochanteric fracture, 35 (35%) received a transfusion, and of those diagnosed with a femoral neck fracture, 29 (27.8%) received a transfusion of one or more units of packed red blood cells. This difference was not statistically significant (Chi-squared test; *p* = 0.32; Table 2 ).

**Table 2:** Comparison of transfusion requirement data in the different surgical groups

Surgical groups	Transfusion		
	No	Yes	
Pertrochanteric fracture	65	35	100
	65.0%	35.0%	100.0%
Femoral neck fracture	52	20	72
	72.2%	27.8%	100.0%
Total	117	55	172
	68.0%	32.0%	100.0%

**Notes:** Chi-squared test = 1.00; *p* = 0.32.

A reserve of 328 units of packed red blood cells was requested for 167 surgeries, representing 97% of the cases, and 112 units were transfused in 55 patients, or 31.9% of the cases. The IT was calculated at 0.65. The C:T was of 2.9 ( Table 3 ).

**Table 3:** Comparison of the units of packed red blood cells reserved and the units that would be reserved using the proposed protocol

Hemoglobin level (g/dL)	n	Number of units transfused	Number of units reserved	Number of units reserved according to the protocol
≥11	111	34	219	0
9–10.9	49	55	88	49
8–8.9	7	12	12	14
7– 7.9	3	7	5	9
≤6.9	2	4	4	0
Total	172	112	328	72

**Fig. 2:**
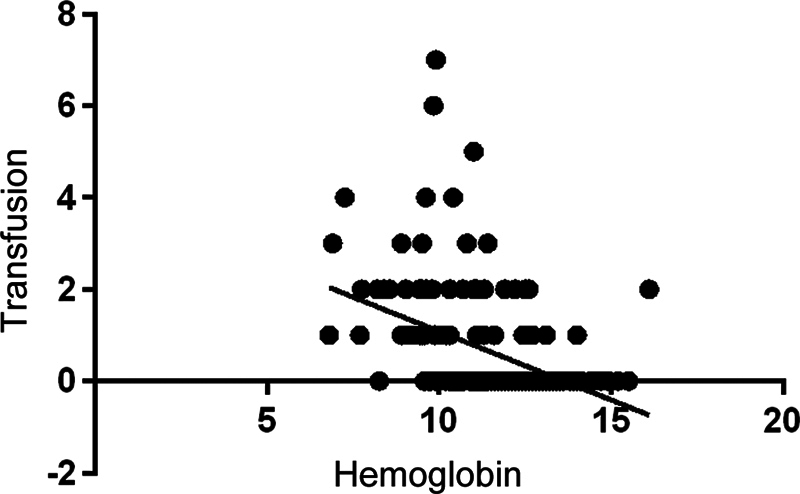
Correlation between preoperative hemoglobin levels (g/dL) and the number of bags transfused.

**Fig. 3:**
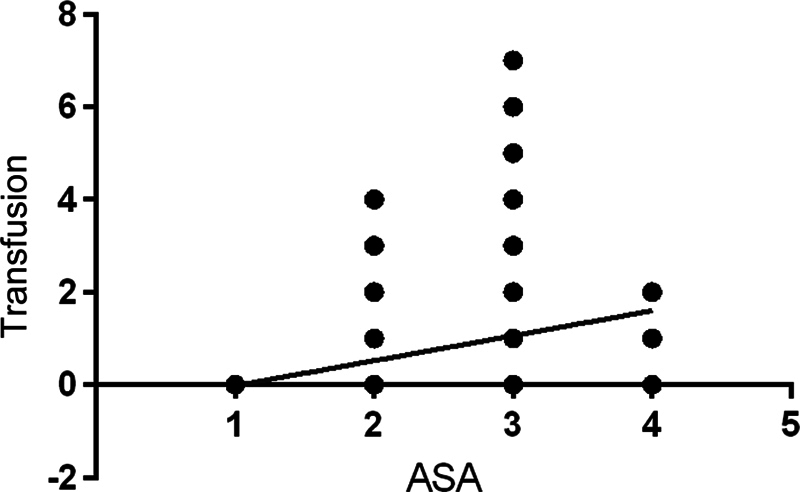
Correlation between anesthetic risk classification (ASA) and the number of bags transfused.

There was a correlation between the anesthetic risk classification (ASA) and the number of transfused bags, according to the Pearson correlation (r = 0.23; *p* = 0.003; Fig. 2 ). There was also an inverse correlation between the preoperative hemoglobin level and the number of bags transfused (Pearson correlation: r = - 0.43; *p* = 0.001; Fig. 3 ).

## Discussion

In line with other studies, the ASA classification and the preoperative hemoglobin level were predictive factors of blood transfusion, since the groups were matched regarding anthropometric characteristics.

The average hemoglobin level on admission was of 11.44 g/dL, and 61 patients (32.4%) had less than 11 g/dL on admission, which characterized a large proportion of patients who had already been admitted with anemia. We must consider the possibility that this is related to the fact that most of the patients are elderly individuals living in low-income families, and that factors related to the food security of these patients may be present. The average drop in hemoglobin after surgery was of 1.54 g/dL; there was no difference in hemoglobin results between patients with a pertrochanteric fracture and those with a femoral neck fracture.

The observed TI of 0.65 indicates that the surgical treatment of these fractures requires requesting a reserve of packed red blood cells. Still, the C:T of 2.9 indicates that the practice of routinely requesting blood reserves for all patients who will undergo hip fracture surgery has proved inefficient, and that it is necessary to adopt some criteria for the best use of these resources.

Khan et al. 14 proposed a protocol to request blood for the surgical treatment of hip fractures ( Table 1 ). In a simulation in which we used these criteria for our sample ( Table 2 ), we obtained a C:T of 0.64, which would indicate efficiency in the reserve request. We also observed that if this protocol had been used, analyzing only individuals with a hemoglobin level greater than or equal to 11, we would have obtained a TI of 0.30. According to these same authors, 14 surgeries with a TI lower than 0.5 do not require a request for a packed red blood cell reserve, thus reducing the fear of not requesting a blood reserve for this group of patients.

The limitation of the present study is that it is a retrospective cohort study from a single hospital. Its primary clinical implication is to demonstrate that requesting packed red blood cells indiscriminately for all patients with hip fractures is unjustified, and that adopting the proposed protocol can safely save resources.

In addition to the financial impact, the request for blood reserves without criteria creates an operational burden. The crossmatch test and IAS take up around 40 minutes of the blood bank's time.

## Conclusion

We found a correlation between the ASA classification and transfusion. Applying a proposed protocol to decide between requesting a reserve and just typing for these patients proved suitable for our casuistry.
