# Supplementary material for: Evaluation of a Blood Reserve Protocol for Hip Fracture Surgery in the Elderly
Source: Rev Bras Ortop (Sao Paulo). 2024 Sep 4;59(4):e617–21. doi: 10.1055/s-0044-1785520 (PMC11374402; doi:10.1055/s-0044-1785520)
Supplement: Related texts

# Research article: Avaliação de um protocolo para reserva de sangue para cirurgias de fratura do quadril em idosos

**Keywords:** fraturas do quadril, tipagem sanguínea, transfusão sanguínea

## Abstract

**Objetivo** Identificar os fatores preditivos da necessidade de transfusão durante e após a cirurgia para o tratamento de fratura do quadril em idosos e avaliar um protocolo para orientar a solicitação de reserva de sangue para a cirurgia.

**Métodos** Foram levantados os prontuários de 172 pacientes idosos submetidos a tratamento cirúrgico de fratura proximal do fêmur. Os dados relativos a sexo, idade, nível pré-operatório de hemoglobina, diagnóstico e tipo de cirurgia foram testados para a verificar a existência de correlação com a transfusão sanguínea. Determinamos o número de unidades reservadas de concentrado de hemácias, o índice de transfusão e a razão prova cruzada:transfusão em nossa casuística, e fizemos os mesmos cálculos em uma situação hipotética em que a solicitação de reserva de sangue para a nossa amostra seguisse os critérios de um protocolo definido.

**Resultados** Encontramos correlação entre a classificação da American Society of Anesthesiologists (ASA) e a ocorrência de transfusão, e correlação inversa entre o nível de hemoglobina de entrada e o número de bolsas transfundidas. Foi solicitada reserva de 328 unidades de concentrado de hemácias para 167 cirurgias. Caso se tivesse aplicado o protocolo proposto, teriam sido solicitadas 72 unidades para 61 cirurgias.

**Conclusão** Encontramos correlação entre as variáveis classificação da ASA e nível pré-operatório de hemoglobina e a ocorrência de transfusão. A aplicação de um protocolo proposto para decidir entre a solicitação de reserva e apenas a tipagem para indivíduos idosos que serão submetidos a tratamento cirúrgico de fratura do quadril mostrou-se adequada para diminuir o número de reservas de concentrado de hemácias.

## Introdução

As fraturas do quadril no idoso são um problema de saúde pública em todo o mundo. Além de estarem associadas a altas taxas de morbimortalidade, geram impacto financeiro importante para os sistemas de saúde. 1

Diversos fatores que influenciam na evolução desses pacientes são estudados, entre eles, a importância de um cuidado multidisciplinar, 2 o tempo transcorrido entre a internação e a cirurgia, 3 o problema do uso prévio de anticoagulantes, 4 os tipos de implantes utilizados 5 etc. Entre essas questões, o gerenciamento da utilização de hemoderivados para o tratamento da anemia pré e pós-operatória tem um peso relevante. A transfusão de sangue faz parte do arsenal terapêutico no cuidado clínico do idoso com fratura do quadril, mas é um procedimento associado a riscos, como aumento das taxas de infecção pós-operatória 6 e do custo do tratamento.

Os critérios para a indicação de transfusão de concentrado de hemácias em idosos submetidos a tratamento cirúrgico de fratura do quadril ainda não estão bem definidos. Existem protocolos liberais que indicam transfusão quando os níveis de hemoglobina caem abaixo de 10g/dL, e protocolos restritivos que não indicam transfusão a menos que o nível de hemoglobina caia abaixo de 8 g/dL, ou que o paciente tenha sintomas relacionados à anemia. Existem evidências de que os protocolos liberais não apresentam vantagens no que se refere à mortalidade, à recuperação funcional e às complicações quando comparados a protocolos restritivos, o que indica que o protocolo restritivo pode ser considerado como melhor opção. 7

Outras questões importantes são em qual situação e qual a quantidade de concentrado de hemácias solicitar para pacientes que serão submetidos ao tratamento cirúrgico. A solicitação de reserva de concentrado de hemácias implica a realização de três procedimentos pelo banco de sangue: a tipagem ABO e Rh, a prova de antígeno inespecífico (PAI) e as provas cruzadas de compatibilidade entre amostras do doador e do receptor. 8 9 Portanto, cada reserva de bolsa de sangue solicitada representa um impacto financeiro e gera demanda de trabalho para o banco de sangue.

Nem todos os pacientes submetidos a tratamento cirúrgico de fratura do quadril necessitam receber transfusão de hemocomponentes; alguns estudos citam incidência em torno de 30% a 40%. 7 10 11 12 Portanto, conhecer os fatores preditivos da necessidade de transfusão de concentrado de hemácias pode orientar a solicitação de sangue antes da cirurgia. Para os pacientes identificados com baixo risco de transfusão durante e após a cirurgia, a solicitação apenas da tipagem sem a solicitação de reserva representaria um avanço importante na gestão de recursos escassos, como são os hemoderivados.

Este estudo tem como objetivo identificar fatores preditivos da necessidade de transfusão durante e após a cirurgia para tratamento de fratura do quadril em idosos e avaliar um protocolo para orientar a solicitação de reserva de sangue para a cirurgia.

## Materiais e Métodos

## Procedimentos Éticos

Este projeto foi aprovado pelo Comitê de Ética em Pesquisa institucional sob o número CAAE: 70915423.1.0000.0099.

## Delineamento e População de Estudo

Esta coorte retrospectiva foi desenvolvida a partir de prontuários eletrônicos dos pacientes com mais de 60 anos submetidos a tratamento cirúrgico de fratura da região proximal do fêmur em nossa instituição, de 1 ^o^ de janeiro a 31 de dezembro de 2021, para a coleta dos dados. As informações relativas à solicitação e à utilização foram fornecidas pelo Instituto de Hematologia da nossa instituição. Foram excluídos pacientes com fraturas por traumas de alta energia e aqueles submetidos à fixação percutânea com parafusos canulados.

## Coleta de Dados

Os dados relativos a sexo, idade, nível pré-operatório de hemoglobina, diagnóstico e tipo de cirurgia foram testados para a verificar a existência de correlação com a transfusão sanguínea.

Foram calculados o índice de transfusão (IT) e a razão prova cruzada:transfusão (C:T). 13 14 O IT é a medida da média de unidades de sangue utilizada por procedimento, e valores menores do que 0,5 indicam que a solicitação rotineira de reserva de sangue não é necessária para o procedimento. A C:T é a razão entre o número de unidades de sangue solicitadas e o número de unidades transfundidas, e é uma medida da eficiência da solicitação de sangue. Valores acima de 2,5 indicam solicitação excessiva. 13

Com base nesses critérios, Khan et al. 14 propuseram um protocolo para a solicitação de sangue para o tratamento cirúrgico das fraturas do quadril ( Tabela 1 ). Inserimos neste protocolo os dados obtidos em nossa amostra para verificar a sua aplicabilidade em nossa casuística por meio da avaliação dos resultados obtidos para a C:T, do número de transfusões realizadas sem que se houvesse pedido a reserva, e do IT para cada intervalo de nível de hemoglobina do protocolo.

**Tabela 1:** Protocolo para solicitação de sangue para cirurgias de fratura do quadril proposto por Khan et al. 14

Nível de hemoglobina (g/dL)	Solicitação de sangue
≥11	Tipagem
9,0–10,9	Reserva de 1 unidade
8,0–8,9	Reserva de 3 unidades
7,0–7,9	Reserva de 4 unidades
≤6,9	Avaliação clínica

## Análise Estatística

Para análise estatística, utilizou-se o programa PASW Statistics for Windows (SPSS Inc., Chicago, IL, Estados Unidos), versão 18.0, com intervalo de confiança de 95% e nível de significância de 5% ( *p* < 0,05) para os testes aplicados. O teste de Shapiro-Wilk foi usado para verificar a normalidade dos dados, e o teste *t* de Student pareado, para a comparação dos níveis pré e pós-operatórios de hemoglobina nos dois grupos de fraturas (pertrocantéricas e do colo do fêmur).

## Resultados

Foram coletadas informações de 172 prontuários de pacientes que se enquadravam nos requisitos do estudo ( Tabela 1 ). Nossa amostra foi composta por 118 pacientes do sexo feminino e 54 do sexo masculino, e a média de idade foi de 80,2 anos. O diagnóstico foi de fratura pertrocantérica em 100 casos, e de fratura do colo de fêmur em 72.

O nível médio de hemoglobina no pré-operatório foi de 11,44 g/dL, e no pós-operatório, de 9,9 g/dL. Os níveis pré-operatórios de hemoglobina foram de 11,0 g/dL e 11,7 g/dL para as fraturas pertrocantéricas e do colo do fêmur, respectivamente. Na classificação da American Society of Anesthesiologists (ASA), 3 pacientes foram classificados como ASA 1, 128, como ASA 2, 38, como ASA 3, e 3, como ASA 4. Após a cirurgia, a média foi de 8,9 para as pertrocantéricas e de 9,2 para as do colo do fêmur. Embora tenha sido observada uma redução na hemoglobina dos pacientes no pós-operatório (teste *t* pareado; *p* = 0,0001), não se verificaram diferenças estatísticas entre os grupos de fraturas (teste *t* não pareado; *p* = 0,17; Fig. 1 ).

**Fig. 1:** Variação da hemoglobina (g/dL) nos diferentes grupos cirúrgicos.

Dos indivíduos com diagnóstico de fratura pertrocantérica, 35 (35%) receberam transfusão, e daqueles com diagnóstico de fratura do colo do fêmur, 29 (27,8%) receberam transfusão de 1 ou mais concentrado de hemácias. Essa diferença não foi estatisticamente significativa (Teste do qui-quadrado; *p* = 0,32; Tabela 2 ).

**Tabela 2:** Comparação dos dados da necessidade de transfusão nos diferentes grupos cirúrgicos

Grupos cirúrgicos	Transfusão		
	Não	Sim	
Fratura pertrocantérica	65	35	100
	65.0%	35.0%	100.0%
Fratura do colo fêmur	52	20	72
	72.2%	27.8%	100.0%
Total	117	55	172
	68.0%	32.0%	100.0%

**Nota:** Teste do qui-quadrado = 1,00; *p* = 0,32.

Foi solicitada a reserva de 328 unidades de concentrado de hemácias para 167 cirurgias, o que representa 97% dos casos, e foram transfundidas 112 unidades em 55 pacientes, ou 31,9% dos casos. O IT foi calculado em 0,65. A C:T foi de 2,9 ( Tabela 3 ).

**Tabela 3:** Comparação da quantidade de unidades de concentrado de hemácias efetivamente reservada e da quantidade que seria reservada na utilização do protocolo proposto

Nível de hemoglobina	n	Número de unidades transfundidas	Número de unidades reservadas	Número de unidades reservadas segundo o protocolo
≥11	111	34	219	0
9–10,9	49	55	88	49
8–8,9	7	12	12	14
7–7,9	3	7	5	9
≤6,9	2	4	4	0
Total	172	112	328	72

Verificou-se uma correlação entre a classificação do risco anestésico (ASA) e o número de bolsas transfundidas, segundo a correlação de Pearson (r = 0,23; *p* = 0,003; Fig. 2 ). Além disso, verificou-se correlação inversa com o nível pré-operatório da hemoglobina e o número de bolsas transfundidas (correlação de Pearson: r = - 0,43; *p* = 0,001, Fig. 3 ).

**Fig. 2:** Correlação entre os níveis pré-operatórios de hemoglobina (g/dL) e o número de bolsas transfundidas.

**Fig. 3:** Correlação entre a classificação do risco anestésico (ASA) e o número de bolsas transfundidas.

## Discussão

Em conformidade com outros estudos, a classificação da ASA e o nível pré-operatório de hemoglobina foram fatores preditivos de transfusão sanguínea, uma vez que os grupos são pareados em relação às características antropométricas.

O nível médio de hemoglobina na admissão foi de 11,44 g/dL, e 61 pacientes (32,4%) tinham menos do que 11 g/dL na internação, o que caracteriza uma grande proporção de pacientes que já foram internados com anemia; há que se considerar a possibilidade de haver relação com o fato de que a maioria dos pacientes são idosos que vivem em famílias de baixa renda, e que fatores relacionados à segurança alimentar destes pacientes possam estar presentes. A queda média da hemoglobina após a cirurgia foi 1,54 g/dL; não houve diferença nos resultados de hemoglobina entre os pacientes com fratura pertrocantérica e os com fratura do colo do fêmur.

O IT observado de 0,65 indica que o tratamento cirúrgico dessas fraturas requer a solicitação de reserva de concentrado de hemácias, mas a C:T de 2,9 indica que o costume de solicitar reserva de sangue rotineiramente para todos os pacientes que serão submetidos a cirurgia por fratura do quadril mostrou-se ineficiente, e que é necessária a adoção de algum critério para a melhor utilização destes recursos.

Khan et al. 14 propuseram um protocolo para solicitação de sangue para o tratamento cirúrgico das fraturas do quadril ( Tabela 1 ). Em uma simulação em que utilizamos esses critérios para a nossa amostra ( Tabela 2 ), obtivemos uma C:T de 0,64, o que indicaria eficiência na solicitação de reserva. Observamos também que caso o referido protocolo tivesse sido empregado, analisando apenas os indivíduos com nível de hemoglobina maior ou igual a 11 g/dL, teríamos obtido um IT de 0,30. Segundo esses mesmos autores, 14 cirurgias com IT menor do que 0,5 não necessitam de solicitação de reserva de concentrado de hemácias, o que diminui, portanto, o receio de não solicitar reserva de sangue para esse grupo de pacientes.

Este estudo tem como limitação ser um estudo de coorte retrospectivo de um único hospital. Sua implicação clínica principal é demonstrar que a prática de solicitar reserva de concentrado de hemácias indiscriminadamente para todos os pacientes com fratura no quadril não se justifica, e que a adoção do protocolo proposto pode proporcionar economia de recursos com segurança.

Além do impacto financeiro, a solicitação sem critério de reserva de sangue gera uma sobrecarga operacional. A prova cruzada de compatibilidade e a PAI consomem cerca de 40 minutos de trabalho do banco de sangue.

## Conclusão

Encontramos correlação entre a classificação da ASA e transfusão. A aplicação de um protocolo proposto para decidir entre a solicitação de reserva e apenas a tipagem para esses pacientes mostrou-se adequada para nossa casuística.
